# Supplementary material for: The Relationship between Diabetes Mellitus Type II and Intervertebral Disc Degeneration in Diabetic Rodent Models: A Systematic and Comprehensive Review
Source: Cells. 2020 Sep 29;9(10):2208. doi: 10.3390/cells9102208 (PMC7600368; doi:10.3390/cells9102208)
Supplement: Supplementary file 1 [file cells-09-02208-s001.pdf]

Review

The Relationship between Diabetes Mellitus Type II and Intervertebral Disc Degeneration in Diabetic Rodent Models: A Systematic and Comprehensive Review

Mohamed Mahmoud [1], Maria Kokozidou [1], Alexander Auffarth [2] and Gundula Schulze-Tanzil [1,*]

[1] Department of Anatomy Paracelsus Medical University, Nuremberg and Salzburg, 90419 Nuremberg, Germany; mmagdibayoumi@gmail.com (M.M.); maria.kokozidou@pmu.ac.at (M.K.); gundula.schulze@pmu.ac.at (G.S.-T.)

[2] Department of Orthopedics and Traumatology, Paracelsus Medical University, 5020 Salzburg, Austria; a.auffarth@salk.at (A.A.)

* Correspondence: gundula.schulze@pmu.ac.at; Tel.: +49-(0)-911-398-6772

Received: 31 July 2020; Accepted: 23 September 2020; Published: date

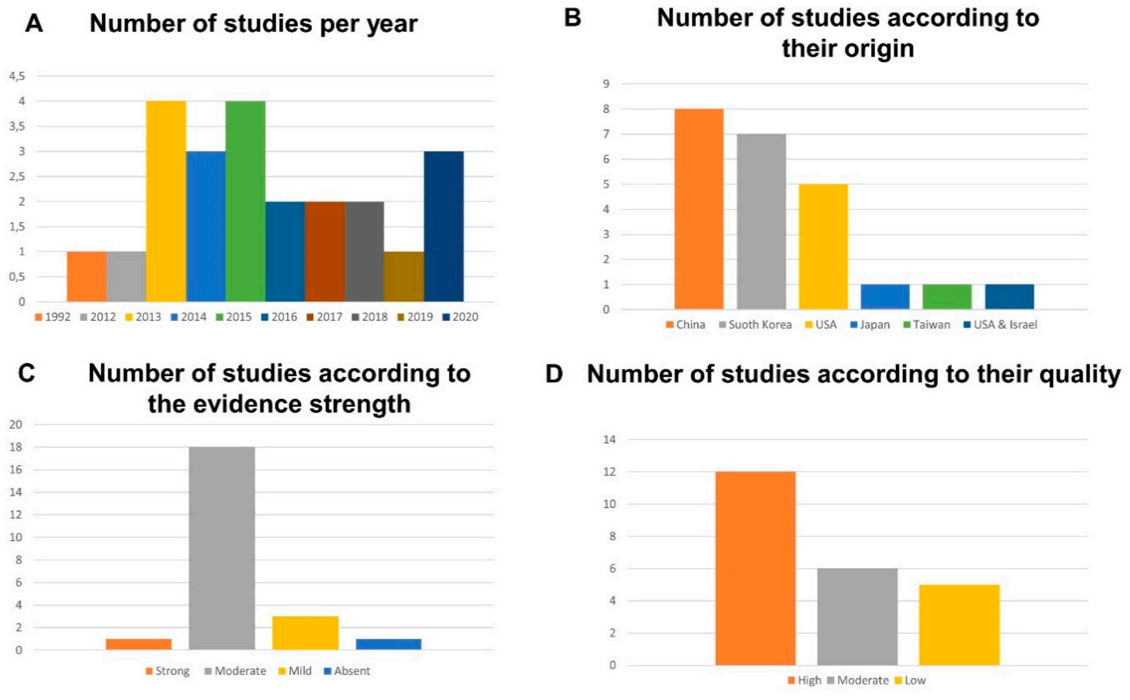

Figure S1: Included studies evaluated in regard to publication date (**A**), origin (**B**), evidence strength (**C**), and quality (**D**).
